# Pratique de la mesure ambulatoire de la pression artérielle à Brazzaville (Congo): données préliminaires

**DOI:** 10.11604/pamj.2015.20.353.6591

**Published:** 2015-04-14

**Authors:** Stéphane Méo Ikama, Bernice Mesmer Nsitou, Jospin Makani, Bertrand Ellenga-Mbolla, Louis Igor Ondze-Kafata, Solange Flore Mongo-Ngamami, Mûnka Nkalla-Lambi, Thierry Raoul Gombet, Gisèle Kimbally-Kaky

**Affiliations:** 1 Département de Médecine, Faculté des Sciences de la Santé de l'Université Marien Ngouabi de Brazzaville, Congo; 2 Service de Cardiologie, Centre Hospitalier Universitaire de Brazzaville, Congo; 3 Service des urgences, Centre Hospitalier Universitaire de Brazzaville, Congo

**Keywords:** Hypertension artérielle, holter tensionnel, Congo

## Abstract

**Introduction:**

Évaluer l'apport de la MAPA dans la prise en charge de l'hypertension artérielle à Brazzaville.

**Méthodes:**

Cette étude transversale descriptive a été menée à Brazzaville entre janvier 2011 et décembre 2013 (soit 36 mois). Elle a inclus une série consécutive de 1040 patients ayant bénéficié d'une Mesure Ambulatoire de la Pression Artérielle. Nous avons utilisé le TONOPORT V et le logiciel Cardiosoft 6.51 de GE Health Care, respectivement pour l'enregistrement et l'analyse des données. Les seuils fixés sur les moyennes de 24H étaient une PA < 130/80 mmHg pour les patients contrôlés, et une PA > 130/80 mmHg pour la confirmation de l'HTA.

**Résultats:**

Il s'agissait de 573 hommes (55%) et de 467 femmes (45%), âgés en moyenne de 51,7 ± 10,6 ans (extrêmes: 22 et 89 ans). L'indication de la MAPA était à visée thérapeutique dans 627 cas (60,3%), à visée diagnostique dans 410 cas (39,4%), et dans trois cas une suspicion d'effet « blouse blanche ». Dans l'indication à visée diagnostique, l'HTA était confirmée dans 303 cas (74%). La moyenne nycthémérale était de 139 ± 12 mmHg pour la PAS et 89,7 ± 9,6 mmHg pour la PAD; 141,2 ± 13,9 mmHg de PAS et 92,4 ± 10,0 mmHg de PAD en période diurne; 131,1 ± 13,5 mmHg de PAS et 80,7 ± 9,9 mmHg de PAD en période nocturne. Dans l'indication à visée thérapeutique, l'HTA était contrôlée chez 220 patients (35%). La moyenne nycthémérale était de 139 ± 14 mmHg pour la PAS et 88,1 ± 10 mmHg pour la PAD. Les moyennes diurnes et nocturnes étaient respectivement de 140,7 ± 14,0 mmHg et 133,1 ± 16,2 mmHg pour la PAS, 90,3 ± 10,5 et 81,1 ± 10,9 mmHg pour la PAD. Le protocole antihypertenseur utilisé était une monothérapie dans 126 cas (22%), une bithérapie dans 270 cas (47%), une trithérapie dans 149 cas (26%), une quadrithérapie et plus dans 29 cas (5%).

**Conclusion:**

Cette étude préliminaire a montré l'importance de la MAPA comme outil de diagnostic et d’évaluation thérapeutique. Son utilisation rationnelle dans notre contexte permettrait d'améliorer la prise en charge des patients hypertendus.

## Introduction

L'hypertension artérielle (HTA) est un problème majeur de santé publique en Afrique Sub-saharienne (ASS), du fait de sa prévalence sans cesse croissante, et d'une surmortalité due essentiellement à l'accès limité aux soins [1]. La prise en charge de cette HTA reste difficile dans notre contexte, en raison de certaines particularités: risque génétique plus important, survenue précoce et souvent sévère, nombreuses complications à la fois cardiaque, cérébrale et rénale. Les données épidémiologiques disponibles montrent des taux de prévalence très variables d'un pays à l'autre [1]. Au Congo, la prévalence de l'HTA était estimée à 32,5% à Brazzaville [2]. Les recommandations actuelles des sociétés savantes mettent un accent particulier sur les mesures ambulatoires par rapport à la mesure en clinique, pour le diagnostic et la surveillance des patients hypertendus traités, en raison de certaines variétés particulières telles que l'HTA « blouse blanche » et l'HTA « masquée » [3, 4]. Le présent travail, dont le but est d'améliorer la prise en charge des patients hypertendus à Brazzaville, s'est fixé pour objectif d’évaluer l'apport de la Mesure Ambulatoire de la Pression Artérielle (MAPA) dans le management au quotidien de l'HTA à Brazzaville.

## Méthodes

Il s'est agi d'une étude transversale descriptive, à recueil de données prospectif, réalisée à Brazzaville entre le 1^er^ janvier 2011 et le 31 décembre 2013 (soit 36 mois). Elle a inclus une série consécutive de 1040 patients, ayant bénéficié d'une MAPA, soit à visée diagnostique d'HTA, soit à visée thérapeutique (évaluation de traitement) pour des patients hypertendus connus et traités régulièrement depuis au moins six semaines. Nous avons utilisé le TONOPORT V et le logiciel Cardiosoft 6.51 de GE Health Care, respectivement pour l'enregistrement et l'analyse des données. Les seuils de normalité fixés étaient une pression artérielle (PA) < 130/80 mmHg pour les moyennes de 24 heures; PA < 135/85 mmHg pour les moyennes diurnes (7h - 22h); PA < 125/80 mmHg pour les moyennes nocturnes (22h ‘ 7h). Le seuil retenu pour la confirmation du diagnostic d'HTA était une PA ‘ 135/85 mmHg sur les moyennes diurnes; et l'HTA était considérée comme contrôlée pour une PA < 130/80 mmHg sur l'ensemble des moyennes de 24 heures. Les patients non-dippers étaient définis comme ceux dont la baisse de la pression nocturne par rapport aux moyennes diurnes était inférieure à 10%, ou qui présentaient une élévation nocturne de la pression artérielle.

Plusieurs variables ont été étudiées: sociodémographiques (âge, sexe, niveau d'instruction, niveau de vie); l'indication de la MAPA; la nature du médecin prescripteur; Les moyennes de pression artérielle (24h, diurnes, nocturnes); le protocole antihypertenseur utilisé, et les classes thérapeutiques utilisées. Le niveau d'instruction comportait quatre modalités (primaire, secondaire, supérieur, aucun), et le niveau de vie trois (faible, moyen, élevé) sur la base de l’étude de pauvreté au Congo (ECOM). Les données, présentées sous forme de proportions pour les variables qualitatives, de moyennes ± écarts-types pour les variables quantitatives, ont été traitées et analysées avec les logiciels Epi-Info 3.5.1 et SPSS 11.1. Pour les comparaisons, nous avons utilisé le test de Khi-2 pour les variables qualitatives et l'analyse des variances (ANOVA) pour les variables quantitatives. Le seuil de significativité a été fixé à p < 0,05.

## Résultats

Les 1040 patients se répartissaient en 573 hommes (55%) et 467 femmes (45%), âgés en moyenne de 51,7 ± 10,6 ans (extrêmes: 22 et 89 ans), avec une différence significative entre les deux sexes (52,5 ± 10 versus 50,7 ± 11,2 ans; p = 0,006). Les patients, sans aucun niveau d'instruction dans 104 cas (10%), avaient un niveau d'instruction primaire dans 228 cas (22%), secondaire dans 550 cas (52,8%), et supérieur dans 158 cas(15,2%). Ils étaient d'un niveau de vie faible dans 416 cas (40%), moyen dans 485 cas (46,6%), et élevé dans 139 cas (13,4%). Le Tableau 1 présente les principales caractéristiques de la population d’étude. L'examen était prescrit par un cardiologue dans 920 cas (88,5%), un médecin généraliste dans 52 cas (5%), un neurologue dans 31 cas (3%), un endocrinologue dans 23 cas (2,2%), et un néphrologue dans 10 cas (1%). Les principales indications de la MAPA étaient un examen à visée diagnostique dans 410 cas (39,4%), à visée thérapeutique dans 627 cas (60,3%); et dans trois cas (0,3%), il s'agissait d'une suspicion d'effet « blouse blanche ». Dans l'indication à visée diagnostique, l'HTA était confirmée dans 303 cas (74%). La moyenne nycthémérale était de 139 ± 12,6mmHg pour la pression artérielle systolique (PAS) et 89,7 ± 9,6 mmHg pour la pression artérielle diastolique (PAD); 141,2 ± 14mmHg de PAS et 92,4 ± 10 mmHg de PAD en période diurne; 131,1 ± 13,5 mmHg de PAS et 80,7 ± 10 mmHg de PAD en période nocturne. Dans l'indication à visée thérapeutique, l'HTA était contrôlée chez 220 patients (35%). La moyenne nycthémérale était de 139 ± 14 mmHg pour la PAS et 88,1 ± 10 mmHg pour la PAD. Les moyennes diurnes et nocturnes étaient respectivement de 140,7 ± 14mmHg et 133,1 ± 16,2 mmHg pour la PAS; 90,3 ± 10,5 et 81,1 ± 11 mmHg pour la PAD. Chez ces 627 patients, 267 (42,5%) étaient identifiés comme des non-dippers. Le protocole antihypertenseur utilisé était une monothérapie dans 126 cas (22%), une bithérapie dans 270 cas (47%), une trithérapie dans 149 cas (26%), une quadrithérapie et plus dans 29 cas (5%).

**Tableau 1:** Caractéristiques de la population d’étude

	Patients (N= 1040)
Hommes	573 (55%)
Age moyen (ans)	51,7 ± 10,6 (22 & 89)
Niveau d'instruction secondaire	550 (52,8%)
Niveau de vie moyen	485 (46,6%)
MAPA à visée diagnostique	410 (39,4%)
MAPA à visée thérapeutique	627 (60,3%)
Suspicion d'effet « blouse blanche »	3 (0,3%)
MAPA 24h (PAS/PAD) diagnostique (mmHg)	139 ± 12,6 / 89,7 ± 9,6
MAPA 24h (PAS/PAD thérapeutique (mmHg)	139 ± 14 / 88 ± 10
Bithérapie	270 (47%)
Quadrithérapie et plus	29 (5%)
Patients non-dippers	267 (42,5%)

MAPA: Mesure Ambulatoire de la Pression Artérielle; PAS: pression artérielle systolique; PAD: pression artérielle diastolique

## Discussion

La Mesure Ambulatoire de la Pression Artérielle (MAPA), technique non invasive, est une méthode de mesure de la pression artérielle (PA) qui établit une bonne corrélation entre le niveau des chiffres de PA et l'atteinte des organes cibles, le risque de maladie cardiovasculaire, et le pronostic à long terme des patients, à la différence des mesures en clinique conventionnelles [4]. Aussi, elle est reconnue comme le gold standard dans le diagnostic de la vraie hypertension artérielle [5]. Il s'agit d'un outil recommandé chez des sujets à haut risque cardiovasculaire, et ceux nécessitant une baisse nocturne de la PA comme les sujets âgés et obèses, ceux porteurs d'une HTA secondaire ou résistante, les diabétiques, les sujets présentant un syndrome métabolique ou un syndrome d'apnée du sommeil [4, 5]. En Afrique Subsaharienne, peu de travaux basés sur la MAPA ont été rapportés [6]. Ceci peut s'expliquer en partie par la faible disponibilité de cet outil dans la plupart de nos structures sanitaires, mais aussi par la probable méconnaissance de l'importance de cette méthode par les médecins ayant au quotidien la gestion des patients hypertendus. En effet, dans notre étude, il ressort que la MAPA était prescrite dans près de 9 cas sur 10 par un cardiologue, avec une faible proportion de médecins généralistes qui constituent le premier maillon de la chaîne de prise en charge de l'HTA. Ce constat a été relevé par l’équipe d'Abidjan [6] avec 98% de prescription de la MAPA par les cardiologues. Ceci témoigne donc de la nécessité de vulgariser cette technique auprès des médecins généralistes et des autres spécialistes ayant en charge des patients hypertendus au quotidien. Dans notre étude, les indications de la MAPA se répartissaient entre l'examen à visée thérapeutique (évaluation du traitement) et l'examen à visée diagnostique (suspicion d'HTA) dans respectivement 60 et 40% des cas. Dans la série abidjanaise [6], les indications étaient essentiellement dominées par la suspicion d'HTA (81,4%), avec une très faible proportion pour l’évaluation thérapeutique (4,3%).

Dans l'indication à visée diagnostique, l'hypertension artérielle a été confirmée dans 3/4 cas. Ainsi donc, près de 25% des patients avec suspicion d'hypertension avaient un profil tensionnel normal, ce qui, en l'absence de la MAPA, aurait pu conduire à la mise en route de façon abusive d'un traitement antihypertenseur pour de « fausses HTA ». Dans l'indication à visée thérapeutique, le contrôle tensionnel était obtenu chez plus d'un patient sur trois. Ce taux de 35% de patients à l'objectif tensionnel, loin d’être satisfaisant, apparaît tout de même élevé en comparaison de celui de 24,6% rapporté dans un travail antérieur [7], et de ceux très variables rapportés dans d'autres pays africains [8, 9] allant de 2,6% au Nigéria à 45,8% au Burkina-Faso; la quasi-totalité de ces études s’étant basé sur la seule mesure en clinique dont on connaît actuellement les limites [10–15]. En effet, les recommandations actuelles des sociétés savantes mettent un accent particulier sur l'importance des mesures en dehors du cabinet, c'est-à-dire l'automesuretensionnelle et la MAPA dans la prise en charge des patients hypertendus à la fois à visée diagnostique et thérapeutique [4, 5]. Certaines de ces études ont mis en exergue la supériorité et la fiabilité de la MAPA par rapport à la mesure en clinique sur les notions de contrôle tensionnel, d'HTA « masquée » et d'HTA « blouse blanche ». Parmi ces différentes études, une plus récente [16] a été particulièrement illustrative. Dans cette dernière, il ressortait que chez des patients hypertendus traités et apparemment contrôlés, sur la mesure en clinique, près des 2/3 ne sont finalement pas à l'objectif tensionnel lorsqu'ils bénéficient d'une MAPA, témoignant ainsi d'une proportion non négligeable d'HTA « masquée ». Aussi, un patient sur 8 ayant une élévation de la PA en clinique a en fait une HTA « blouse blanche ».

## Conclusion

Cette étude préliminaire a montré l'importance de la MAPA dans la prise en charge des patients hypertendus à Brazzaville. Il s'agit d'un outil diagnostique dans certaines situations particulières (HTA « masquée », HTA « blouse blanche »), mais aussi d’évaluation thérapeutique pour apprécier le niveau de contrôle tensionnel. Dans notre contexte, compte tenu de sa faible disponibilité et de son coût pour ces populations aux conditions sociales souvent modestes, il convient d'en faire un usage rationnel. Aussi, pour améliorer la prise en charge des patients, sa vulgarisation auprès des médecins généralistes et des autres spécialistes ayant en commun la gestion de l'hypertension artérielle paraît nécessaire.
